# Why should the Next Generation of Youth Guidelines Prioritize Vigorous Physical Activity?

**DOI:** 10.1186/s40798-024-00754-0

**Published:** 2024-07-30

**Authors:** Helmi Chaabene, Adrian Markov, Lutz Schega

**Affiliations:** 1 Department of Sport Science, Chair for Health and Physical Activity, Otto-von-Guericke University Magdeburg, Magdeburg, Germany; 2 Université de Jendouba, Institut Supérieur de Sport et de l’Education Physique du Kef, Le Kef, 7100 Tunisie; 3 Independent researcher, Potsdam, Germany

**Keywords:** Physical Activity Recommendations, Children, Adolescents, Physical Activity Intensity, Adherence rate, Physical Fitness, Health

## Abstract

The health benefits of regular physical activity (PA) in youth are well-documented. Yet the adherence rate to PA guidelines among youth worldwide is alarmingly deficient with only 19% of youth worldwide adhering to the World Health Organization guidelines. This is reflective of a global proliferation of a physical inactivity pandemic among youth. The negative consequences of physical inactivity on health are profound, as they threaten to persist into adulthood, exacerbating the burden of preventable health issues. There is persuasive evidence that vigorous PA generates physical fitness and health benefits surpassing those of low- or moderate-intensity activity in youth. In addition, indications show that the adherence rate to vigorous PA among youth exceeds that relative to for low- or moderate-intensity activity. As a result, promoting vigorous PA can help mitigate the global issue of low adherence to PA in youth. Therefore, in this Current Opinion paper, we argue that vigorous PA, compared to low or moderate-intensity activity, holds greater significance for youth’s health and physical fitness. Additionally, the potential implications derived from the existing evidence regarding vigorous PA on the current guidelines for youth are discussed.

## Key Points

The health benefits of regular physical activity in youth are well-established. Yet the adherence rate to physical activity guidelines among youth worldwide is alarmingly deficient with around 80% of youth worldwide not adhering to the World Health Organization guidelines.

Persuasive evidence derived from observational, controlled interventions, as well as narrative and systematic reviews with meta-analysis, indicate that vigorous physical activity generates positive effects exceeding those induced by low- or moderate-intensity activity on youth health and physical fitness.

The next generation of physical activity recommendations should place a greater emphasis on the significance of vigorous physical activity for youth.

## Introduction

The health benefits of regular physical activity (PA) in youth (an umbrella term for children and adolescents) are well-established [1, 2]. Notably, regular PA holds the potential to enhance bone health, maintain a healthy weight, improve cardiorespiratory and muscular fitness, support cardio-metabolic health, enhance cognitive function, and reduces the risk of depression, among others [1]. The guidelines set forth by the World Health Organization (WHO) for youth recommend an average of 60 min of moderate-to-vigorous, mostly aerobic PA daily with additional muscle and bone strengthening exercises carried out at least three times weekly [2]. Yet the adherence rate to PA guidelines among youth worldwide is alarmingly deficient with 81% of youth aged between 11 and 17 years failing to adhere to the WHO guidelines, rendering them insufficiently physically active [3]. This troubling trend underscores the global proliferation of a physical inactivity pandemic among youth individuals [4] with immediate (e.g., reduced cardiorespiratory fitness, muscle weakness, increased stress and anxiety) as well as long-term (e.g., increased risk of chronic diseases and obesity, decreased bone health, increased risk of depression) negative consequences on physical fitness and health [5–7]. The negative consequences are profound, as this trend threatens to persist into adulthood, exacerbating the burden of preventable health issues [5–7].

It is well-known that youth enjoy PA [7]. However, youth are nowadays living in an environment full of the so-called “sedentary alternatives” [8] where elementary PAs such as cycling and walking have become less common, and safety concerns have restricted playing in the street [7]. Although by nature youth like to play actively, the sedentary alternatives that have arisen over the past two decades due to technological advancements seem to hold greater appeal for young people than PA [7]. Unfortunately, sedentary alternatives are now part of current life and cannot be completely avoided. Sedentary alternatives are linked to severe adverse health consequences. For example, there is compelling evidence that higher durations as well as more frequent exposure to television viewing and screen time are associated with numerous adverse health outcomes such as unhealthy body composition and higher cardiometabolic risks [9]. Additionally, increased screen time is associated with lower physical fitness and self-esteem [9]. In the USA, the television viewing of children averaged 3.5 ± 4.0 h per day, representing almost one-third of the waking time [7].

While the last WHO guidelines related to PA in youth are evidence-based with some details pertaining to the amount and types of PA that generate significant health benefits and alleviate potential health risks, they remain relatively vague. In particular, the specific PA dose in terms of volume, intensity, and frequency leading to the best possible health and physical fitness benefits is as yet unknown [10]. More specifically, there is accumulating evidence that vigorous PA is more beneficial for the promotion of youth health than moderate-intensity activity [11–22]. Earlier studies revealed that every minute of vigorous PA leads to the same improvements in adiposity as 2–3 min of moderate PA in youth [23, 24]. Additionally, it has been revealed that vigorous PA in the form of high-intensity interval training (HIIT) is more effective than low-to-moderate intensity PA for enhancing cardiorespiratory fitness in overweight and obese youth [22]. More specifically, HIIT resulted in greater benefits on maximal oxygen uptake (VO_2max_) than low-to-moderate intensity PA (effect size = 0.59; 1.92 ml/kg/min) [22]. Furthermore, a recent systematic review with meta-analysis showed that cardiometabolic risk score was inversely correlated (*r* = -0.13; 95% confidence interval [CI] = -0.24 to -0.02) with vigorous PA and consistently associated with cardiorespiratory fitness (*r* = 0.25; 95%CI = 0.15 to 0.35) in youth [11]. These findings support the need to emphasize vigorous over low- and moderate-intensity PA in youth.

Furthermore, unlike in adults [4, 25], vigorous PA is more attractive for youth. In fact, youth prefer to engage in short, intermittent high-intensity efforts rather than low-intensity ones [26, 27]. Therefore, promoting vigorous PA could improve the adherence rate to PA and mitigate the current physical inactivity pandemic. Considering all the above, we advocate in this Current Opinion paper that the intensity of PA is more appealing than the volume for youth health and physical fitness. Further elaboration on the importance of the intensity (i.e., quality) of PA compared to its volume (i.e., quantity) on health and fitness-based outcomes from the available literature will be provided. Furthermore, the potential implications of the available evidence related to vigorous PA on the adherence rate and the current PA guidelines for youth will be discussed.

## Historical Background of Physical Activity Guidelines in Youth

The acknowledgment that an appropriate amount of PA is crucial for health dates back to at least the 5th century B.C. [28]. In modern times, precisely in the 20th century, the American College of Sports Medicine (ACSM) published the first PA guidelines, albeit for adults [29]. In 1988, the ACSM published its first set of youth guidelines indicating that youth should obtain 30 min of vigorous exercise daily [30]. A first consensus statement for adolescents from the 1993 International Conference on Physical Activity Guidelines indicated that adolescents should be physically active daily or nearly every day as part of their lifestyle activities [31]. The recommendations also stressed the fact that adolescents should perform 3 or more 20 min sessions of moderate-to-vigorous PA each week [31]. Two years later, a joint recommendation by the US Centers for Disease Control and Prevention and the ACSM in 1995 advocated all children and adults accumulate a minimum of 30 min daily of moderate-intensity PA [32]. The recommendation also highlighted that greater health benefits might be expected by increasing either the duration or the intensity of PA [32].

The first significant effort providing specific recommendations for PA in youth was published in the 1996 Surgeon General’s Report on Physical Activity and Health in the United States [33]. The recommendations indicate that all people above the age of two years should accumulate at least 30 min of moderate-intensity endurance-type PA, on most or preferably all days of the week [33]. The same report also pointed to the fact that engaging in more vigorous PA can lead to more health and functional benefits. In addition to endurance type PA, it was recommended to perform strengthening exercises at least twice per week, including the major muscle groups [33]. In 2010, the WHO released its first detailed PA guidelines indicating that youth should engage in at least 60 min daily, moderate-to-vigorous, mostly aerobic PA [34]. The recommendations further indicated that vigorous-intensity aerobic activities in addition to those that strengthen the muscles and bones should be performed at least 3 times per week [34]. The last WHO update in 2020 [2] was very similar to the previous. The only difference related to the wording change from “at least” 60 min to “an average of” 60 min of moderate-to-vigorous PA per day given that this was considered to better reflect the body of the available evidence and the way moderate-to-vigorous PA has been quantified [2].

## Thresholds for and Adherence to Physical Activity Guidelines

### Thresholds for Moderate and Vigorous Physical Activity

It is worth noting that there is no consistent standard for the vigorous PA cutpoint [35]. Nevertheless, it is accepted that vigorous PA refers to activities performed at an intensity ≥ 80% maximum heart rate [36], between 75 and 80% of peak oxygen uptake (VO_2peak_) [37], at 6.0 or more metabolic quivalents of task (METs) [38], that corresponds to a 7 or 8 on a subjective rating of perceived exertion (RPE) scale of 0–10 [2, 39], or that is > 4000 counts per minute using an accelerometer [11, 40]. Moderate PA on the other hand stands for activities that are performed at an intensity between 55 and 60% VO_2peak_ [37], between 3 and < 6 METs [38], that correspond to an RPE around 5 or 6 on a scale of 0–10 [2, 39], or that is between 2296 and 4011 counts per minute using an accelerometer [40].

### Adherence to Physical Activity Guidelines

Despite the well-established benefits of PA, accumulated evidence indicates that the adherence rate among youth worldwide is alarmingly low [3, 41]. In a study including 1.6 million youth aged 11 to 17 years from 146 countries, Guthold et al. [3] reported that 81% were not adhering to the PA guidelines and were therefore considered insufficiently active. The same authors reported a sex disparity, with girls exhibiting a relatively higher prevalence of physical inactivity (84.7%) compared to boys (77.6%) [3]. Similarly, recent evidence from a systematic review with meta-analysis including 3.3 million participants across 32 countries indicated an adherence rate to aerobic and muscle strength activities approaching 20% among adolescents aged 12 to 17 years [41]. Likewise, a very high level of physical inactivity, ranging from 67 to 73%, has been reported among youth aged between 5 and 17 years across 49 countries [42]. In this same context, Hallal et al. [43] studied the PA levels in adolescents from 105 different countries and revealed that four-fifths of them do not achieve the recommended guidelines. Overall, these results highlight a notably low adherence rate to the recommended PA and therefore, are of much concern. As such, all stakeholders are urged to act and elaborate a different comprehensive and effective strategy to overcome the current physical inactivity pandemic among youth worldwide.

## Track of Physical Activity from Youth to Adulthood

The decrease in PA seems to take place early in life [44, 45]. Additionally, there is compelling evidence that PA behavior tracks from childhood to adolescence [46] and adulthood [5, 6, 47, 48]. For example, Thompson et al. [49] investigated the influence of PA levels during childhood and adolescence on adult PA attitudes and behavior. They reported that negative early PA experiences (e.g., inadequate skill development, size disadvantage, or less attention from teachers/coaches) were associated with decreased adult PA levels [49]. Conversely, adults who remained physically active recalled positive experiences, including parental support and positive influences from friends and teachers/coaches [49]. Telama et al. [50] conducted a 27-year longitudinal study involving 3,596 Finnish children aged 3–18 years. Their findings indicated that PA behavior established during childhood tends to persist into adulthood with a moderate-to-high stability coefficient (≥ 0.60). Jose et al. [51] examined the factors that impact PA behavior during the transition from adolescence to adulthood. They revealed that perceived sports competency in females was associated with being regularly active during the transition from adolescence to adulthood. In males, cardiorespiratory fitness, playing sports outside school, and having active fathers during childhood and adolescence were associated with regular activity during this transition. Likewise, there is evidence that the level of physical fitness at young ages facilitates the recognition of those at increased risk of adopting a sedentary behavior during adulthood [52]. Tammelin [53] conducted a narrative review of the literature and revealed a positive correlation between youth and adult PA. Although the exact value of the correlation was not reported, the review indicated that PA tends to track from youth into adulthood. The same author recommended engaging early in PA to increase the chances of keeping physically active at later ages of life [53]. Similarly, there is evidence that regular participation in organized youth sports increases the likelihood of adopting a physically active lifestyle during adulthood [48]. Moreover, earlier studies demonstrated that physical fitness tracks from childhood and adolescence to adulthood (*r* = 0.38 to 0.51) [5, 6]. Altogether, there is strong evidence that PA and physical fitness track from youth to adulthood, emphasizing the relevance of adopting an active lifestyle at early ages to increase the likelihood of keeping physically active at later ages. This is of utmost importance for health during youth and adulthood alike [2, 7].

## The Effects of Vigorous versus Low-to-Moderate Physical Activity on Health Factors

### Body Composition

Abbott and Davies [54] indicated that the time spent in vigorous, but not in moderate, PA was associated with a lower percentage of body fat in children aged 8 years (*r* = -0.44). Further, Wittmeier et al. [23] showed that 45 min daily of moderate PA was needed to obtain the same benefits as only 15 min daily of vigorous PA in terms of reduced body fat and body mass index (BMI). Likewise, Steele et al. [24] revealed that 6.5 min of daily vigorous PA was associated with a 1.32 cm reduction in waist circumference while 13.6 min daily of moderate PA would be needed to reach a waist circumference reduction of 0.49 cm. This would mean that 2 to 3 min of moderate PA would be needed to generate similar benefits of only 1 min of vigorous PA [55]. In a comprehensive narrative review of the literature, Owens et al. [55] addressed the health-related benefits of vigorous compared to moderate or low PA in youth. They concluded that vigorous aerobic PA leads to positive effects on adiposity in youth above those obtained from moderate or low aerobic PA. In a systematic review with meta-analysis of controlled trials, Costigan et al. [56] revealed moderate positive effects of HIIT on body composition (i.e., BMI [effect size = -0.37; 95%CI = -0.68 to -0.05] and body fat percentage [effect size = -0.67; 95%CI = -1.30 to − 0.0.04) of youth aged between 13 and 18 years. García-Hermoso et al. [11] conducted a systematic review with meta-analysis of the prospective correlation between vigorous PA and health-related factors in youth. The pooled data from 21 studies indicated that vigorous PA seems to be negatively associated (*r* = -0.09; 95%CI = -0.15 to -0.03) with adiposity among youth at follow-up [11]. Overall, there are consistent results that vigorous PA generates positive effects on body composition beyond the effects induced by moderate or low PA. In addition, vigorous PA is time-efficient compared to low or moderate PA, making it more appealing for youth [56].

### Cardiometabolic Health

Carson et al. [16] conducted a two-year prospective cohort study to assess the effects of PA intensity on cardiometabolic risk factors in youth and demonstrated that the time spent in vigorous PA was associated with improved cardiometabolic risk factors (i.e., cardiorespiratory fitness, waist circumference, systolic blood pressure, BMI). Hay et al. [20] examined the association between PA intensity and cardiometabolic risk factors in youth and reported that only vigorous PA was consistently associated with lower levels of waist circumference, BMI, and systolic blood pressure in youth (*r* = -0.11 to -0.19). In another study [35], substituting low-intensity PA with vigorous PA was shown to be associated with 0.67 to 7.30 cm smaller waist circumference and 12.6 to 27.0 pmol/L lower insulin values in youth. Moreover, the results of an experimental study conducted with youth [21] showed that 7 weeks of either moderate or vigorous PA improve cardiometabolic risk factors (e.g., BMI and aerobic fitness [ES = 0.68 to 2.29]). The same authors reported some distinct adaptations such as decreased systolic blood pressure (ES = 0.65) after vigorous PA and reduced percentage of body fat (ES = 0.72) and improved insulin sensitivity (ES = 0.80) following moderate PA [21]. Notably, the time commitment over the 7-week training period was 420 min for the moderate PA group and only 63 min for the vigorous PA group [21]. This reflects the time efficiency of vigorous PA given that significant improvements in cardiometabolic risk factors occurred with just 15% of the moderate exercise time [21]. Racil et al. [57] investigated the effects of 12 weeks of moderate versus HIIT on selected cardiometabolic markers in obese youth aged 16 years. They demonstrated that high- but not moderate-intensity interval training decreased waist circumference (∆-3.5%), triglyceride (∆-5.3%), and total cholesterol levels (∆-7%) [57]. The results of a recent systematic review with meta-analysis of the prospective correlation between vigorous PA and health-related factors in youth indicated that vigorous PA is negatively associated with cardiometabolic risk score among youth at follow-up (*r* = -0.13; 95%CI = -0.24 to -0.02) [11]. To sum up, there is persuasive evidence that vigorous PA generates positive effects exceeding those induced by moderate PA on cardiometabolic health in youth.

### Bone Health

The available evidence points towards greater effects of vigorous weight-bearing activities (referred to as “high-impact” activities in bone health studies) on bone health than moderate (“low-impact”) weight-bearing activities in youth [55, 58]. Sayers et al. [59] revealed that vigorous (bootstrap path coefficient = 0.082; 95%CI = 0.037 to 0.128 [Bootstrap path coefficient represents standard deviation change per doubling of vigorous PA]) but not moderate PA was positively associated with tibial cortical bone mass in youth. Likewise, there is evidence that vigorous but not low or moderate PA predicts femoral neck strength in youth, explaining 5 to 9% of its variance [60]. More specifically, the authors showed that at least 25 min of vigorous PA daily was associated with better femoral neck bone health [60]. Weeks et al. [61] conducted a randomized controlled trial investigating the effect of 10 min jumping activity on bone health in youth. They demonstrated significantly more bone mass at the femoral neck, trochanter, and whole body than in controls after 8 months of training (11.6% vs. 5.6%, 14.9% vs. 4.6%, and 8.6% vs. 5%, respectively, for the experimental and control groups) [61]. Similar results were found in another randomized controlled trial after 16 months of vigorous PA training in youth [62]. García-Hermoso et al. [11] conducted a systematic review with meta-analysis of the prospective correlation between vigorous PA and health-related factors in youth and revealed that vigorous PA is positively correlated with total body bone mineral density among youth at follow-up (*r* = 0.16; 95%CI = 0.06 to 0.25) [11]. While there is no definitive evidence-based dosage for optimizing bone health benefits, recommendations suggest that incorporating vigorous PA, especially weight-bearing exercises with ground reaction forces equivalent to 3 to 5 times one’s body mass, for a minimum of 7 months, with session durations of 1 to 12 min, and a frequency of 2 to 12 sessions per week, can have a positive impact on bone health [63]. In summary, the available data indicate that vigorous PA, such as weight-bearing and jump exercises, has a more favorable impact on bone health in youth when compared to moderate or low PA.

### Brain Health

There is evidence that PA interventions positively impact mental health in youth, irrespective of the intensity [64, 65]. However, there are some indications in the literature that vigorous PA is associated with better cognitive performance compared to moderate PA. For instance, Coe et al. [66] showed that higher amounts of vigorous (first semester: chi-squared = 10.1, *p* < 0.05; second semester: chi-squared = 6.05, *p* < 0.05) but not moderate PA were associated with higher grades among sixth-grade students. Further, engaging in PA may improve brain structure and function in youth [36]. These improvements appear to be due to enhanced white matter integrity and greater activation of regions crucial for cognitive processes [36]. In summary, there is consistent evidence that PA has a positive impact on cognitive function and mental health, regardless of its intensity. While there are hints that vigorous PA may produce even more pronounced effects than moderate activity, further exploration of this aspect is warranted in future studies.

## The Effects of Vigorous versus Low-to-Moderate Physical Activity on Physical Fitness

### Cardiorespiratory Fitness

Evidence from observational studies indicates that vigorous PA is associated with increased cardiorespiratory fitness, while moderate activity is not. For example, Hay et al. [20] showed that cardiorespiratory fitness increased in a dose-response pattern across tertiles of vigorous PA (highest tertile = 8.7 ± 1.5 min/day, VO_2max_ = 51 ml/kg/min vs. lowest tertile = 1.4 ± 0.4 min/day, VO_2max_ = 46 ml/kg/min, *p* < 0.05), while low and moderate PA of various amounts were not related with cardiorespiratory fitness in youth. In the same vein, the prospective study by Carson et al. [16] showed that follow-up aerobic capacity increased in a dose-response fashion within quartiles of vigorous PA (VO_2max_ = 43.3 ml/kg/min vs. 50.2 ml/kg/min for quartile 1 and quartile 4, respectively), whereas such a trend was not observed within quartiles of moderate PA among youth. Unlike observational studies, controlled interventions demonstrated comparable gains in aerobic capacity following moderate and vigorous PA in youth [57, 67–69]. For instance, Corte de Araujo et al. [69] contrasted the effect of 12 weeks of HIIT versus continuous endurance training on cardiorespiratory fitness (VO_2max_) in obese youth, reporting similar benefits (∆14.6% and 13.1%, respectively). The authors also highlighted the time-efficiency advantage of HIIT, as it necessitated 70% less time commitment when compared to continuous endurance training [69]. Similarly, Racil et al. [57] demonstrated that moderate and HIIT were equally effective in improving cardiorespiratory fitness (VO_2peak_) in youth (∆5.2% and 7.7%, respectively). The results of the study by Buchan et al. [68] did not deviate from the above-mentioned findings in that the authors showed similar cardiorespiratory fitness gains following 7 weeks of HIIT or lower-intensity continuous aerobic training in youth (88.78 ± 26.41 and 93.25 ± 23.16 shuttles, respectively). Once again, the authors noted that the high-intensity group achieved improvements in a much shorter time commitment compared with the lower-intensity continuous training group [68].

Both narrative and systematic reviews with meta-analysis support the same conclusion that vigorous PA yields superior effects on cardiorespiratory fitness compared to low or moderate PA. For instance, Parikh and Stratton [38] conducted a narrative review of the literature on the effect of PA intensity on cardiorespiratory fitness in youth. They revealed increased cardiorespiratory fitness among participants who spent more time performing vigorous PA. Recently, García-Hermoso et al. [5] conducted a systematic review with meta-analysis of the prospective correlation between vigorous PA and health-related factors in youth. The results indicated that vigorous PA seems to be positively associated with cardiorespiratory fitness among youth at follow-up (*r* = 0.25; 95%CI = 0.15 to 0.35) [5]. The findings of the systematic review with meta-analysis of controlled trials by Costigan et al. [56] indicated that HIIT generates a large positive effect on cardiorespiratory fitness in youth (ES = 1.05; 95%CI = 0.36 to 1.75), making it a time-efficient training approach.

Overall, there is compelling evidence from observational and systematic reviews with meta-analysis that vigorous PA is more effective for improving cardiorespiratory fitness in youth than low or moderate activity. While the available controlled interventions did not reveal any advantage of vigorous PA over moderate PA, it is worth noting that the time-efficiency aspect of the former distinguishes it notably from the latter.

### Muscular Fitness

Unlike cardiorespiratory fitness, studies addressing the impact of vigorous versus moderate PA on measures of muscular fitness (i.e., muscle strength, muscle power and muscular endurance) are scarce. Martínez-Gómez et al. [70] examined the associations between objectively assessed PA and muscular fitness in adolescents. They revealed that vigorous but not low or moderate PA was significantly associated with increased muscular fitness after controlling for the confounding factors of sex, age, pubertal status, BMI, and cardiorespiratory fitness (β = 0.133; adjusted R^2^ = 0.448). Additionally, the same authors found that youth with high vigorous PA levels displayed muscular fitness levels comparable to youth involved in resistance training [70]. In another study conducted with 4-year-old children, Leppänen et al. [71] showed that the time spent on vigorous PA was associated with greater fat-free mass index. More particularly, the same authors revealed that 5 more min/day of vigorous PA was associated with 200 g better handgrip strength. Likewise, 5 more min/day of vigorous PA was associated with 3 cm longer in the standing long jump test [71]. The results of the systematic review by Smith et al. [72] indicated that muscular fitness is consistently associated with PA, particularly with objectively assessed vigorous PA, in youth. Lesinski et al. [73] conducted a systematic review with meta-analysis of controlled interventions on the effects of resistance training on muscular fitness, among other factors. The authors showed that a high training intensity in the range of 80 to 89% of one-repetition maximum was the most favorable for enhancing muscle strength (ES = 2.52) compared with lower intensities in young athletes. Taken together, muscular fitness seems to better benefit from vigorous PA compared to lower intensities activity in youth.

## Why should Youth Guidelines Prioritize Vigorous Physical Activity?

**Fig. 1:**
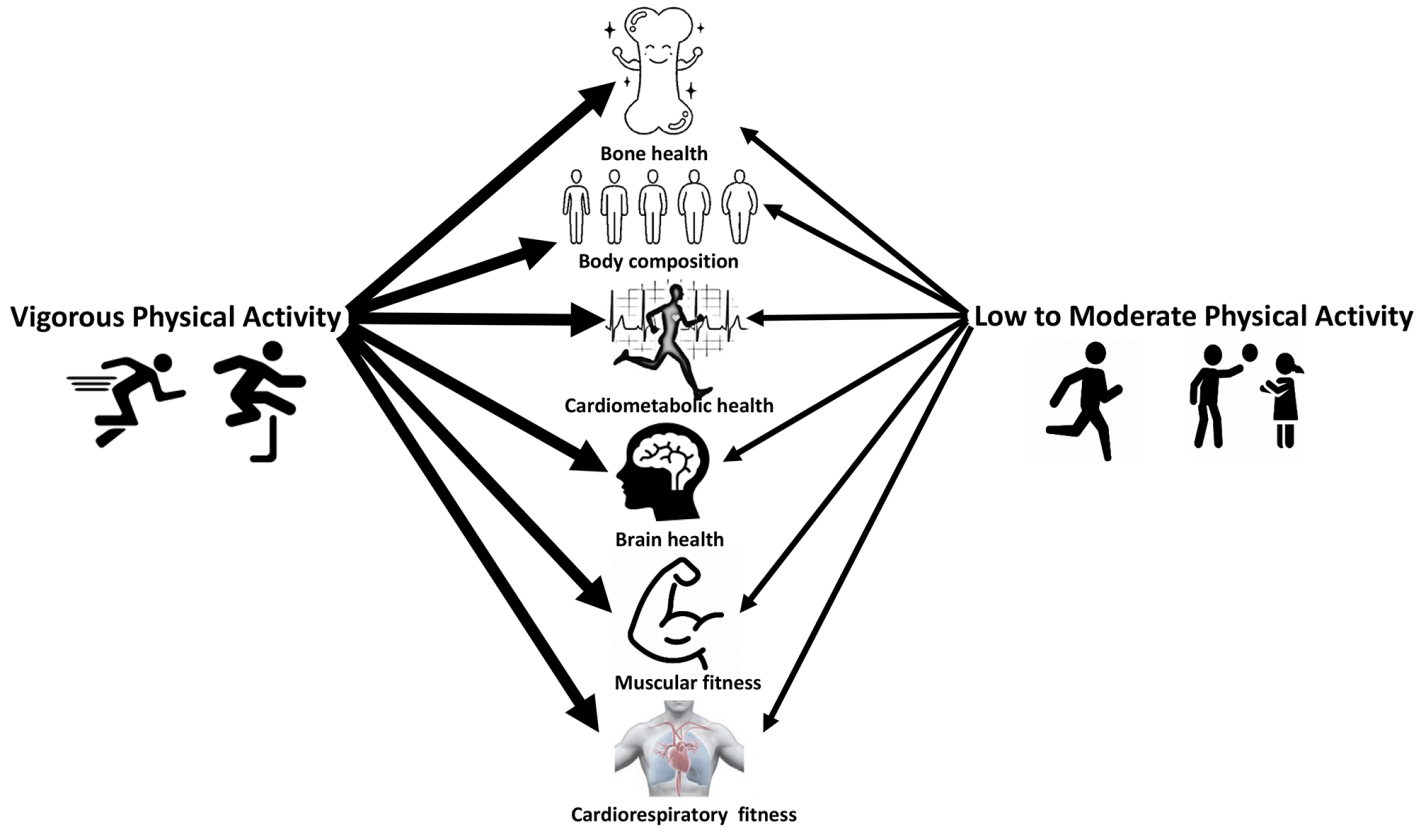
The impact of physical activity intensity on youth health and physical fitness. The larger size of arrows signifies greater benefits

Accumulating evidence indicates that vigorous PA is more appealing than low or moderate activity to improve various physical fitness [38, 55, 70, 73] and health factors [11, 17, 18, 55] in youth **(**Fig. 1**)**. Additionally, vigorous PA is safe with no additional negative health effects compared to moderate PA [68, 74]. Considering the results mentioned above, there is a strong case for promoting vigorous PA. However, the open question remains: what is the optimal amount of vigorous PA? Unfortunately, due to the current lack of randomized controlled studies, this question cannot yet be fully answered. The current PA guidelines in youth [2] are rather general with no clear recommendation as to the exact dose of vigorous PA for optimal physical fitness and health benefits. According to the available evidence, a crude range of vigorous PA doses, spanning from about 10 to 30 min daily, has shown effectiveness in improving health and fitness-related outcomes in youth. Nevertheless, further exploration of this duration range is warranted in future studies. Of note, even among youth who are adhering to the current PA guidelines, the evidence indicated that more time dedicated to vigorous PA was associated with improved health-related outcomes while low PA was negatively associated with health-related outcomes [17]. Overall, given the compelling evidence for the greater benefits of vigorous compared to low or moderate PA for youth health and physical fitness, it seems plausible to favor vigorous over low to moderate PA. In this sense, we advocate that PA guidelines for youth should place greater emphasis on the relevance of vigorous PA. In particular, we propose that the next generation of PA guidelines should prioritize vigorous PA among youth.

## Adherence Rate to Vigorous Physical Activity

Youth PA patterns are well-recognized for their sporadic nature [75], involving daily, brief, and intermittent bouts of high-intensity efforts [26, 27, 39]. Notably, unlike in adults [4, 25, 76], vigorous PA holds a stronger appeal for youth. In fact, youth tend to gravitate towards short, intermittent high-intensity efforts over low-intensity ones [26, 27, 39]. This inclination is substantiated by a study conducted by Hyde et al. [76], which revealed that 70.6% of healthy youth met the recommendations for vigorous PA, while only 15.2% adhered to the guidelines for moderate-to-vigorous PA as per the US Physical Activity guidelines. In obese youth, Corte de Araujo et al. [69] found a similar adherence rate to a 12 weeks HIIT program (3 to 6 sets of 60-sec sprints at maximal effort interspersed with 3 min active recovery) compared to 30–60 min of continuous aerobic exercise at 80% of peak heart rate. More specifically, 86.9% of participants in the HIIT group and 85.5% in the continuous aerobic group completed the program [69]. Additionally, Murphy et al. [77] reported that 7 out of 9 (78%) obese youth completed a 4-week HIIT program compared to 6 out of 8 (75%) in the continuous aerobic training group. Therefore, it is plausible to argue that vigorous PA is more attractive than lower intensities for healthy youth and equally attractive for obese youth. Considering these outcomes collectively, it can be assumed that promoting vigorous PA can help mitigate the significant global issue of low adherence to PA in youth.

## Conclusions and Future Research Perspectives

Persuasive evidence indicates that vigorous PA generates benefits on health and physical fitness that go beyond that of low or moderate PA in youth **(**Fig. 1**)**. In fact, a less time-consuming, more intense dose of PA may be a viable option for youth seeking to achieve greater health as well as physical fitness benefits. As such, we urge national and international health organizations to place greater emphasis on vigorous PA in their next updated guidelines for youth.

Of note, the majority of the existing studies are of observational nature, with only a limited number of controlled interventions. Therefore, future research efforts must prioritize randomized controlled studies to determine the optimal dosage of vigorous PA. Additionally, long-term intervention studies are needed to contrast the effects of vigorous versus low-to-moderate PA on physical fitness and health in youth. This research will help answer questions related to the time course of changes and the long-term benefits of different PA intensities in youth. Another aspect worth mentioning is the inconsistency in defining the thresholds for moderate and vigorous PA [55], implying that a uniform threshold still needs to be established. This would optimize the interpretation of the findings related to the effects of vigorous and low-to-moderate PA. Moreover, whether the association between vigorous PA and health and physical fitness outcomes is moderated by type (e.g., aerobic vs. muscle strengthening), location (outdoor vs. indoor), or domain (e.g., physical education vs. leisure time) of PA is as yet unclear and still needs to be further investigated.

## Data Availability

Not applicable.

## Abbreviations

PA: Physical activity
HIIT: High intensity interval training
WHO: World Health Organization
ACSM: American College of Sports Medicine
VO_2peak_: peak oxygen uptake
MET: Metabolic equivalent
RPE: Rating of perceived exertion
